# Automatic screening of cervical cells using block image processing

**DOI:** 10.1186/s12938-016-0131-z

**Published:** 2016-02-04

**Authors:** Meng Zhao, Aiguo Wu, Jingjing Song, Xuguo Sun, Na Dong

**Affiliations:** School of Electrical Engineering and Automation, Tianjin University, Tianjin, China; School of Medical Laboratory, Tianjin Medical University, Tianjin, China

**Keywords:** Cervical cells screening, SVM, Features selection, Block image processing, Computational complexity

## Abstract

**Background:**

Cervical cancer is the second leading cause of female-specific cancer-related deaths after breast cancer, especially in developing countries. However, the incidence of the disease may be significantly decreased if the patient is diagnosed in the pre-cancerous lesion stage or earlier. In recent years, computer-based algorithms are widely used in cervical cancer screening. Most of the proposed algorithms follow the procedure of segmentation, feature extraction, and then classification. Nevertheless, few of the existing segmentation methods are as flexible and robust as the human visual system, and the complexity of the algorithms makes it difficult for clinical application.

**Methods:**

In this study, a computer-assisted analytical approach is proposed to identify the existence of suspicious cells in a whole slide cervical cell image (WSCCI). The main difference between our method and the conventional algorithm is that the image is divided into blocks with certain size instead of segmented cells, which can greatly reduce the computational complexity. Via data analysis, some texture and color histogram features show significant differences between blocks with and without suspicious cells. Therefore these features can be used as the input of the support vector machine classifier. 1100 non-background blocks (110 suspicious blocks) are trained to build a model, while 1040 blocks (491 non-background blocks) from 12 other WSCCIs are tested to verify the feasibility of the algorithm.

**Results:**

The experimental results show that the accuracy of our method is about 98.98 %. More importantly, the sensitivity, which is more fatal in cancer screening, is 95.0 % according to the images tested in the study, while the specificity is 99.33 %.

**Conclusion:**

The analysis of the algorithm is based on block images, which is different from conventional methods. Although some analysis work should be done in advance, the later processing speed will be greatly enhanced with the establishment of the model. Furthermore, since the algorithm is based on the actual WSCCI, the method will be of directive significance for clinical screening.

## Background

Accounting for around 8 % of total cancer deaths in women, cervical cancer is the second leading cause of female-specific cancer-related deaths after breast cancer [1], yet it can be prevented at its early stage by detection of precancerous cells in smear tests [2]. The most famous success in smear screening is its ability to reduce the mortality and morbidity of cervical cancer. Once the abnormal cells are detected, the patient can be scheduled for further examination and treatment. Consequently, the patient can be cured at an early stage.

Conventionally smear tests are based on microscopic observations to identify abnormalities in the structure and morphology of cells, which may be inconsistent because of subjective variability of different observers [3]. To lower the false negative rate in screening, many advanced technologies and commercial devices have been developed and introduced [4–8]. The Cytoanalyzer project attempted firstly to build an automated screening device for PAP-Smears in 1950s [4]. Unfortunately, tests with the Cytoanalyzer revealed that it produced too many false alarms on the cell level [5]. Since then, it took more than 40 years before the first successful commercial system (developed by Tripath Company) appeared. A new liquid based specimen preparation technique called SurePath has been added to further improve the system performance and it can also analyze conventional smears [6]. The system can be used to recognize about 25 % of the slides as normal for no further review; the other 75 % are ranked into five categories at risk for abnormality [7], further human recognition is needed. As for liquid based preparation technique, Cytyc Company, which later became part of the Hologic Company, takes the leading position. The system is marketed for increasing detection of abnormalities by improved specimen preparation and screening both visually and by machine [8]. But till now automated screening has not been sufficiently cost-effective to completely replace the visual systems judging from the relatively limited penetration of automated screening systems in the screening operations worldwide [9].

The computer-aided image analysis technique used to assist artificial diagnosis of cell abnormalities or tumors can availably reduce the influence of humankind and improve the efficiency and accuracy of screening. Computerized methods have been increasingly evolved in the area of medical image analysis, especially in cells (cell nuclei) detection, segmentation, and classification [10]. Reliability, accessibility, cost, efficiency, and technical maintenance should be taken into account in any new designs.

Segmentation and classification are two main tasks in cervical cell screening, and more and more automatic [11–14] and semiautomatic [15–17] methods have been proposed. For cell segmentation, related works can be divided into two groups: pure nuclei segmentation [18], both cytoplasm and nuclei segmentation [19]. Approaches including thresholding [20], active contour [21], morphology (watershed) [22, 23], graph cuts [17], level-set [19] are most commonly used. It is required for a good cell segmentation method to accurately detect and delineate the cells or cell nuclei under different staining conditions and in the presence of disturbing object in the direct vicinity and dealing with the overlapping problems. Computational efficiency is another important requirement since the whole screening process should be accomplished in an acceptable time. However, no existing method is as flexible and robust as the human visual system in really identifying where the nuclear or cytoplasmic border located in difficult cases [9], and the efficiency still remains a tough task.

The ultimate goal of the screening process is to find the women with precancerous lesions, or find the abnormal cells from the cervical cell images, so that they can be treated before the malignancy develops into potentially lethal invasive cancer. Therefore the classification after segmentation is also a very important procedure, where K-means clustering [24], support vector machine (SVM) classifier [16, 25], AdaBoost [26] and artificial neural networks (ANN) [27] are the most frequently used algorithms. For classification, reliability, efficiency and accuracy are the most important measurement criteria. However, most of the published classification methods are designed for presegmented images that contain only one cell [12], and some are based on the benchmark database presented in [28].

To reach the goal of automatic screening of cervical cells, and to improve the efficiency of screening, instead of segmentation, the block-based classification algorithm is proposed in this study. Firstly, cervical images are divided into blocks of the same size. Basically, there are six kinds of block types, including background blocks, blocks with few white cells, blocks with many white cells, blocks with clustered white cells, blocks with normal epithelial cells and blocks with suspicious epithelial cells. Only the blocks with suspicious epithelial cells are recognized as abnormal blocks. Subsequently, the background blocks are removed to speed up the computation. Then, texture and color features are evaluated by using statistical analysis to select salient variables for designing a classifier to discriminate the suspicious blocks from normal ones. Finally, SVM classifier is used to distinguish normal and abnormal blocks. 1100 non-background blocks, which contain 100 suspicious blocks, are the training set. 491 non-background blocks from 12 other images, which contain 40 suspicious blocks, are the testing set. The SVM classifier is trained with salient attributes selected based on the training set, and tested with the testing set. The rest of the paper is organized as follows. Section “Methods” describes the materials and methods proposed and adopted in this study. Section “Results and discussion” demonstrates the experimental results and brief discussion. Finally, conclusions are made in section “Conclusion”.

## Methods

**Fig. 1:**
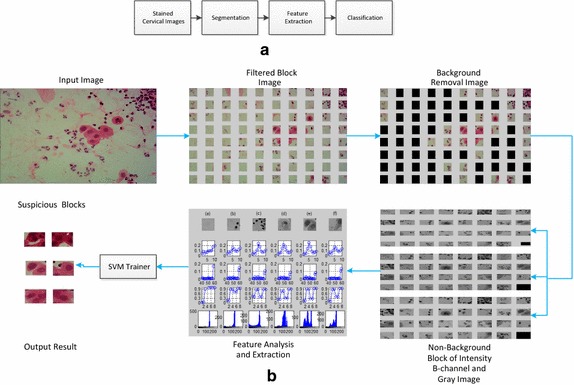
Flowchart of automatic cervical cell screening algorithm. **a** Flowchart of conventional cellular image analysis segmentation is usually the precondition of classification. **b** Flowchart of the algorithm proposed in this paper to improve the accuracy and efficiency, the segmentation is omitted, and the block-based processing algorithm is adopted. The images were divided into blocks, and then features of three color models were analyzed. An SVM classifier was adopted to build a model and to classify the blocks into normal and abnormal. The output was the suspicious blocks

The analysis system is composed of a personal computer, automated cervical images processing and analyzing programs, and SVM classifier. The flowchart of the overall experimental procedure is illustrated in Fig. 1. It can be summarized as: acquisition of block images, background removal, three color models of background blocks, features and parameters selection, classification using SVM and assessment of performance.

### Materials

All the analysis and experiments are based on actual patients’ pathological images with a mix of normal and abnormal cells provided by Medical University of Tianjin from Tianjin Tumor Hospital and stained with hematoxylin and eosin (H&E) [29]. The images are from different individuals. As we just need to do preliminary screening without future distinguishing, H&E staining is appropriate [30]. 1100 non-background block images, including 100 suspicious blocks are the training set. Other 960 blocks, including 491 non-background blocks from 12 different images are the testing images. To make the result more convincing, the images are of various types, like images with lots of white cells, bacillus, piles of cells, both normal and suspicious cells and only normal cells and so on. So the analysis is relatively comprehensive. All images should have the same resolution and magnification. For better feature analysis, the resolution should be a little higher, like 400× in our case.

The merit of computer-aided methods in discriminating abnormal from normal cells has been widely recognized and accepted in cervical screening programs. However, the false negative cases leading to postponement of optimal treatment are discouraging. In the false negative group, approximately two-thirds are related to sampling/preparation errors, i.e., the inflammatory, bloody or mucinous background, and cellular crowds. The other one-third is related to screening errors and interpretive errors causing abnormal cells not being correctly classified [3]. In this study, the cell samples for image analysis are taken from liquid-based prepared smears which can reduce artifacts inherent in the conventional smears, e.g., poor fixation, thick and overlapping groups, obscuring inflammation, blood and mucus, etc.

### Image preprocessing and background removal

#### Acquisition of block images

Recently, although automatic and semiautomatic methods have been proposed for the segmentation of whole slide cervical cell image (WSCCI), there are still some shortcomings. The performance of automatic detection methods is degraded by cell overlapping, saturation and hue of cell images, and artifacts caused by vaginal secretion and blood stain [36]. Segmentation of cervical cells remains a challenging issue. Furthermore, the computational complexity is also an issue for cell segmentation. Therefore, in this study, a block image processing method is proposed to replace segmentation.

The original cervical images in this experiment are all of the same resolution and magnification, which is 2592 × 1944. Firstly, the images are divided into equally sized blocks, and all the subsequent processing is based on blocks instead of whole images. The images are down sampled into the same size (800 × 1000). As we need to find the abnormal cells from the image, the size of the block should be larger than the suspicious cells, but cannot be too large. With some experimental tests, the images are divided into 8 × 10 blocks, and each block has 100 × 100 pixels.

#### Background blocks removal

In the process of block-image features analysis, it is surprised to find that the distributions of the local binary pattern (LBP) [31, 32], especially the rotation invariant uniform LBP (RIU-LBP) are different between background and non-background blocks.

LBP is a simple and understandable texture operator that labels the pixels of an image by thresholding the neighborhood of each pixel and considers the result as a binary number. It is also an effective and Illumination invariant texture operator that can measure and extract local texture information of images. Another important property is its computational simplicity, which makes it possible to analyze images in challenging real-time settings. So it is widely used in pattern recognition nowadays. The LBP can be calculated according to the following equations:

1 $$\begin{aligned} LBP_{P,R} & = \sum\limits_{l = 0}^{P - 1} {s(g_{l} - g_{c} } )2^{l} \\ s(x) & = \left\{ {\begin{array}{*{20}l} 1 \hfill &\quad {if\;x \ge 0} \hfill \\ 0 \hfill &\quad {else} \hfill \\ \end{array} } \right. \\ \end{aligned}$$

where, *P* is the number of the neighboring pixels, *g*_*c*_ is the value of center pixel and *g*_*l*_ is the value of neighboring pixel.

Uniform LBP (U-LBP) is a useful extension to the original operator, which can reduce the length of the feature vector and implement a simple rotation invariant descriptor. This idea is motivated by the fact that some binary patterns occur more commonly in texture images than others. An LBP is called uniform if the binary pattern contains at most two 0–1 or 1–0 transitions. Using uniform patterns, the length of the patterns will be reduced from 256 to 59 (*P* = 3 × 3), and the rotation invariant uniform patterns will be 10. The definitions are as below:

Uniform LBP:

2 $$\begin{aligned} &U(LBP_{P,R} ) = \sum\limits_{l = 1}^{P - 1} {\left| {s\left( {g_{l} - g_{c} } \right) - s(g_{l - 1} - g_{c} )} \right|} \\ &\quad\quad\quad\quad\quad + \left| {s\left( {g_{l - 1} - g_{c} } \right) - s(g_{0} - g_{c} )} \right| \\ & if\;U \le 2,\;\;{\text{Uniform}} \\ \end{aligned}$$

Rotation Invariant LBP:

3 $$LBP_{P,R}^{ri} = \mathop {min}\limits_{0 \le d < N} \left\{ {\sum\limits_{l = 1}^{P} {s\left( {g_{l} - g_{c} } \right)} 2^{{\left[ {\left( {l + d} \right)modN} \right]}} } \right\}$$

RIU-LBP is a combination of U-LBP and RI-LBP.

**Fig. 2:**
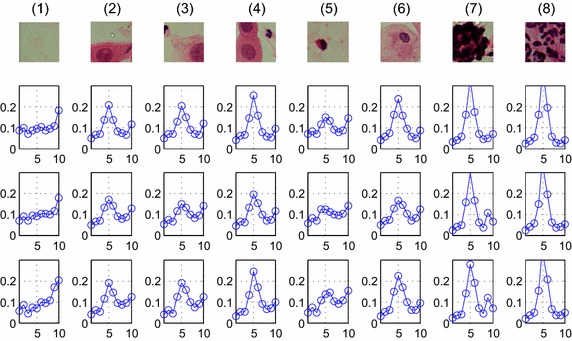
The RIU-LBP features of typical blocks of three color models. The *first row* is the RGB image of eight typical blocks, (**1**) is the background block, (**2**)–(**4**) are the ‘suspicious blocks’, (**5**) the ‘few-white’, (**6**) the ‘normal’, (**7**) ‘clustered white’ and (**8**) ‘many-white’. The *second row* is the RIU-LBP feature of the intensity color model, the *third row* is the RIU-LBP feature of the R-channel color model, and the *last row* is the RIU-LBP feature of the gray color model. The *horizontal axis* represents the ten pattern of RIU-LBP, while the *vertical axis* represents the proportion of each pattern

The RIU-LBPs of typical block images in 3 color models are shown in Fig. 2. In general, the peak of the non-background blocks appears in the fifth, while the background is not, and the accuracy is higher tested with the gray color model. So RIU-LBP features of gray model are used to remove the background.

#### Color models

After the background blocks are removed, the non-background block images are transformed into three color models: R-channel of RGB images, gray images and intensity of HSI model, following the median filtering.

The transformation relations are as follows:

4 $$\left\{ \begin{aligned} R = RGB(:,:,1) \hfill \\ G = RGB(:,:,2) \hfill \\ B = RGB(:,:,3) \hfill \\ \end{aligned} \right.$$

5 $$Gray = 0.299 * R + 0.587 * G + 0.114 * B$$

6 $$\left\{ \begin{aligned} &H = \left\{ \begin{aligned}& \theta ,\;(B \le G) \hfill \\ &360 - \theta ,\;(B > G) \, \hfill \\ \end{aligned} \right. \hfill \\ &S = 1 - \frac{3}{{\left( {R + G + B} \right)}}\left[ {\hbox{min} (R,G,B)} \right] \hfill \\ &I = \frac{1}{3}(R + G + B) \hfill \\ \end{aligned} \right.$$

where, *RGB* is the block image, $\theta = arccos\left\{ {\frac{{\frac{1}{2}\left[ {\left( {R - G} \right) + \left( {R - B} \right)} \right]}}{{\left[ {\left( {R - G} \right)^{2} + \left( {R - G} \right)\left( {G - B} \right)} \right]^{1/2} }}} \right\}$

As the poor contrast, non-uniform staining and noise in cervical cell images will likely have a great impact on the analysis of image characteristics. Image filtering is needed before further steps are taken. It was demonstrated by Tsai et al. [33] that median filter can eliminate both impulse and Gaussian noise in cervical smear images. A 5 × 5 median filter is applied to the original images to discard noise. The formula of median filter is as follow:

7 $$g(x,y) = med\{ f(x - k,y - l),\quad (k,l \in W)\}$$

where, *f*(*x*, *y*) is the source image, *g*(*x*, *y*) is the image after filtering, and *W* = 5 × 5 in our case.

### Feature analysis

**Fig. 3:**
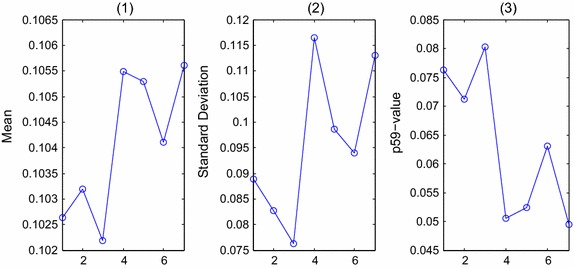
The statistical U-LBP features of typical blocks. The *horizontal axis* in this figure represents seven different types of blocks shown in the first row of Fig. 2(2)–(8). The *vertical axis* in (**1**)–(**3**) demonstrates the mean value, standard deviation value and proportion of pattern 59, respectively

**Fig. 4:**
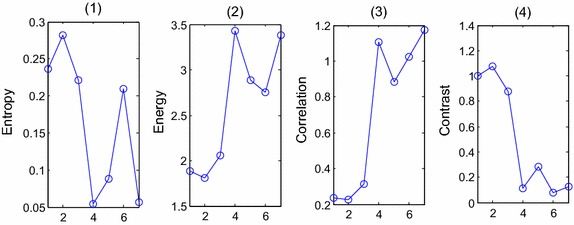
The statistical co-occurrence matrix features of typical blocks. The *horizontal axis* in this figure represents seven different types of blocks shown in the first row of Fig. 2(2)–(8). The *vertical axis* in (**1**)–(**4**) demonstrates Entropy, Energy, Correlation, Contrast, respectively

**Fig. 5:**
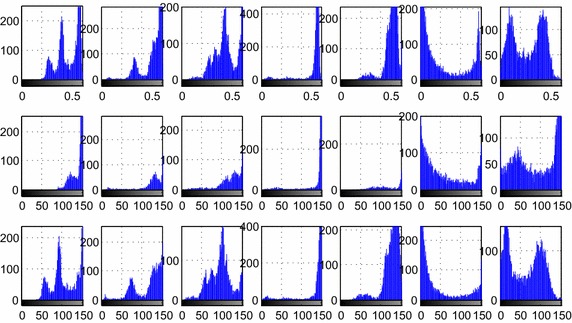
The color histogram of typical blocks in three color models. *Row* (1)–(3) shows the color histogram of Intensity model, R-channel and gray image, respectively, and *column* (1)–(7) represent the seven types of blocks shown in the first row in Fig. 2(2)–(8)

**Fig. 6:**
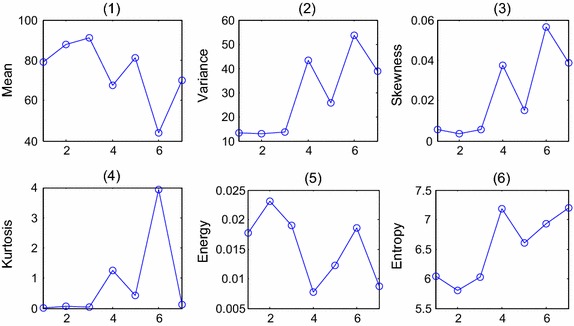
The statistical color histogram features of typical blocks. The *horizontal axis* represents seven different types of blocks shown in the first row in Fig. 2(2)–(8). (**1**)–(**6**) The ‘Mean’, ‘Variance’, ‘Skewness’, ‘Kurtosis’, ‘Energy’ and ‘Entropy’ values, respectively

**Fig. 7:**
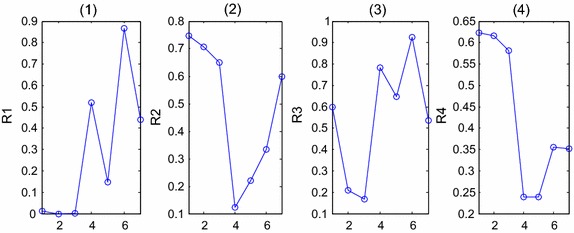
The ratio of intervals. (**1**)–(**4**) Demonstrates the value of four ratios respectively, and the *horizontal axis* represents seven different types of blocks shown in Fig. 2(2)–(8)

Feature analysis is an extra up-front work that should be done before the procedures of feature selection and classification. The most important information about whether the cell is normal or (pre-)malignant is found in the chromatin pattern and texture features of the cells [9]. So texture features, including the U-LBP features [32, 34], RIU-LBP features, co-occurrence matrix features, and color histogram features are analyzed depending on three color models. The first row of Fig. 2 shows the eight examples of different types of blocks, including one background block, three abnormal blocks of different types (‘suspicious blocks’), one block with few white blood cells (‘few-white’), one block with normal cells (‘normal’), one block with clustered white blood cells (‘clustered-white’) and one block with many white blood cells (‘many-white’), and feature figures in the following sections, from Figs. 3, 4, 5, 6 and 7, all depend on these seven non-background blocks.

#### U-LBP features

As described in section “Background blocks removal”, U-LBP contains 59 patterns. Through our analysis, the distributions of patterns 51–59 are different between suspicious blocks and normal blocks. Therefore the mean and standard deviation of the 9 patterns are calculated, together with the proportion of pattern 59, shown in Fig. 3. The horizontal axis in Fig. 3 represents seven different types of blocks shown in the first row of Fig. 2(2)–(8). The vertical axis in Fig. 3(1)–(3) demonstrates the mean values, standard deviation values and proportion of pattern 59, respectively.

#### Attributes of co-occurrence matrix

A statistical method for examining texture is the GLCM, considering the spatial relationship of pixels [35]. It calculates how often a pixel with the intensity (gray-level) value *i* occurs in a specific spatial relationship to a pixel with the value *j*. In this paper, *d* = 2.

8 $$\begin{aligned} P(i,j,d,135^{ \circ } ) & = \# \left\{ {\left( {\left( {k,l} \right),\left( {m,n} \right)} \right) \in \left( {L_{y} \times L_{x} } \right)} \right. \\ & \qquad \times \left( {L_{y} \times L_{x} } \right)|k - m = d,l - n = d, \\ & \qquad or\;(k - m = - d,l - n = - d, \\ & \qquad \left. {I(k,l) = i,I(m,n) = j} \right\} \\ \end{aligned}$$

9 $$\begin{aligned} P(i,j,d,45^{ \circ } ) & = \# \left\{ {\left( {\left( {k,l} \right),\left( {m,n} \right)} \right) \in \left( {L_{y} \times L_{x} } \right)} \right. \\ & \qquad \times \left( {L_{y} \times L_{x} } \right)|(k - m = d,l - n = - d) \\ & \qquad or\; (k - m = - d,l - n = d), \\ & \qquad \left. {I(k,l) = i,I(m,n) = j} \right\} \\ \end{aligned}$$

The GLCM can reveal certain properties about the spatial distribution of the gray levels in the texture image through the following statistics.

Entropy-Measures the uniformity of the probability distribution of the Matrix

10 $$Ent = - \sum\limits_{i = 1}^{k} {\sum\limits_{j = 1}^{k} {P(i,j)\log (P(i,j))} }$$

Energy-Provides the sum of squared elements in the GLCM, also known as uniformity or the angular second moment (ASM)

11 $$Ene = \sum\limits_{i = 1}^{k} {\sum\limits_{i = 1}^{k} {(P(i,j))^{2} } }$$

Correlation–Correlation–Measures the joint probability occurrence of the specified pixel pairs

12 $$Correlation = \sum\limits_{i = 1}^{k} {\sum\limits_{j = 1}^{k} {\frac{(i - \mu i)(j - \mu j)P(i,j)}{{\sigma_{i} \sigma_{j} }}} }$$

Contrast–Measures the local variations in the co-occurrence matrix

13 $$Contrast = \sum\limits_{n = 0}^{k - 1} {n^{2} } \left\{ \begin{aligned} \sum\limits_{i = 1}^{k} {\sum\limits_{j = 1}^{k} {P(i,j)} } \hfill \\ \left| {i - j} \right| = n \hfill \\ \end{aligned} \right\}$$

In this study, the features are calculated in two directions (45°, 135°), and in three color models (Intensity color model, R-channel and gray image).

The features of typical blocks are shown in Fig. 4, the horizontal axis in Fig. 4 represents seven different types of blocks shown in the first row of Fig. 2(2)–(8). The vertical axis demonstrates the Entropy, Energy, Correlation and Contrast values, respectively.

#### Color histogram analysis

Nunobiki et al. [36] reported the usefulness of RGB color specification in analyzing the variation of color proper-ties for Papnicolaou-stained cervical smears. A color histogram is a representation of the distribution of colors in an image. It focuses only on the proportion of the number of different types of colors, regardless of the spatial location of the colors.

14 $$H(i) = \frac{{n_{i} }}{N},\quad i = 0,1, \ldots ,L - 1$$

where, *i*-gray level, *L*-total types of gray level, *n*_*i*_-number of pixels with gray level *i*, *N*-total number of pixels.

The color histogram of typical block is shown in Fig. 5, and the following 6 statistics variables that show the statistical distribution of colors and the essential tone of an image are chosen to quantize the differences between blocks.

Mean–Measures average value of image

15 $$\mu = \sum\limits_{i = 0}^{L - 1} {iH(i)}$$

Variance–Measures how far a set of numbers is spread out

16 $$\sigma^{2} = \sum\limits_{i = 0}^{L - 1} {(i - \mu )^{2} H(i)}$$

Skewness–Measures the asymmetry of the probability distribution of the histogram about its mean

17 $$\mu_{s} = \frac{1}{{\sigma^{3} }}\sum\limits_{i = 0}^{L - 1} {(i - \mu )^{3} H\left( i \right)}$$

Kurtosis–Measures “peakedness” of the probability distribution of the histogram, to judge whether the distribution gathering on the mean

18 $$\mu_{k} = \frac{1}{{\sigma^{4} }}\sum\limits_{i = 0}^{L - 1} {(i - \mu )^{4} H(i)} - 3$$

Energy–Measures the uniformity of the probability distribution of the histogram

19 $$\mu_{N} = \sum\limits_{i = 0}^{L - 1} {H(i)^{2} }$$

Entropy–Also measures the uniformity of the probability distribution of the histogram

20 $$\mu_{e} = - \sum\limits_{i = 0}^{L - 1} {H(i)} log_{2} \left[ {H(i)} \right]$$

The features are calculated in three color models, and the average values are adopted.

The statistic features of different blocks are shown in Fig. 6, and the horizontal axis represents seven different types of blocks shown in the first row in Fig. 2. Figure 7(1)–(6) shows the ‘Mean’, ‘Variance’, ‘Skewness’, ‘Kurtosis’, ‘Energy’ and ‘Entropy’ values respectively.

According to the color histogram in Fig. 5, especially the histogram of Intensity model and gray image, we found that the blocks with abnormal cells usually had 3 peaks, and the pixel numbers in some regions are obviously greater than other blocks. We calculated the ratios of some intervals in Intensity model and gray model, represents as [*R*_1_, *R*_2_, *R*_3_, *R*_4_]. *R*_1_, *R*_2_ are the ratios in Intensity model. *R*_1_ is the ratio between interval [0 0.2] (the intensity interval of white cell nuclear), and interval [0 0.5], and *R*_2_ represents the ratio between interval [0.2 0.4] (the intensity interval of epithelial cell nuclear) and interval [0.2 0.5]. *R*_3_, *R*_4_ are the ratios in gray model. *R*_3_ is the ratio between interval [0 50] (the gray-level interval of white cell nuclear) and interval [0 125], and *R*_4_ is the ratio between interval [50 100] (the gray-level interval of epithelial cell nuclear) and interval [50 125] (the gray-level interval of epithelial nuclear). Figure 7(1)–(4) demonstrate the values of these four ratios, and the horizontal axis represents seven different types of blocks shown in Fig. 2(2)–(8).

### Feature selection

Feature selection has the advantages of reduced number of attributes and the size of storage requirements, decreased computational time and improved predictive performance. In section “Feature analysis”, 17 features consisted of texture (including U-LBP, co-occurrence matrix) and color variables (including histogram statistical and ratio) are analyzed. In this study, statistical analyses of color and textural variables were conducted by *t* test to test significance (p < 0.01) of individual variables. Variables that reach the significance were selected as salient features to design a classifier to detect suspicious from normal blocks.

In section “U-LBP features”, U-LBP features are analyzed. From Fig. 3, the mean and standard deviation value can be adopted. The values are calculated in three color models, and we will take the average values of three color models as the first set of salient variables, symbolized as L.

21 $$\begin{aligned} L & = \left[ {std,p_{59} } \right] \\ std & = \frac{1}{3}(std\_I + std\_R + std\_g) \\ p_{59} & = \frac{1}{3}\left( {p_{59\_I} + p_{59\_R} + p_{59\_g} } \right) \\ \end{aligned}$$

where, *std*_*I*, *std*_*R*, *std*_*g* represent the standard deviations in three color models. *p*_59_*I*_, *p*_59_*R*_, *p*_59_*g*_ represent the proportions of pattern 59 of 3 color models.

In section “Attributes of co-occurrence matrix”, the attributes of co-occurrence matrix are analyzed. According to Fig. 4, the differences of attributes ‘Energy’, ‘Correlation’ and ‘Contrast’ are larger between ‘normal blocks’ and ‘suspicious blocks’, therefore, these 3 variables are chosen as the second set of salient variables, symbolized as C.

22 $$\begin{aligned} C & = [ene,cor,con] \\ ene & = \frac{1}{3}(ene\_I + ene\_R + ene\_g) \\ cor & = \frac{1}{3}(cor\_I + cor\_R + cor\_g) \\ con & = \frac{1}{3}(con\_I + con\_R + con\_g) \\ \end{aligned}$$

where, $ene\_I = \frac{1}{2}(ene\_I^{45} + ene\_I^{135} )$, and the same with the other 8 variables.

In section “Color histogram analysis”, the statistical features and ratios of color histogram are analyzed. According to Fig. 5, four features including $\sigma ,\mu_{s} ,\mu_{N} ,\mu_{e}$ are chosen as the third set of salient variables, symbolized as S.

23 $$S = [\overline{{\sigma^{2} }} ,\overline{{\mu_{s} }} ,\overline{{\mu_{N} }} ,\overline{{\mu_{e} }} ]$$

The average values of three color models will be calculated to get the variables.

According to Fig. 7, the differences of *R*_2_, *R*_4_ are higher between ‘normal blocks’ and ‘suspicious blocks’, so these two features are chosen as the last set of salient variables, symbolized as R.

24 $$R = [R_{2} ,R_{4} ]$$

Therefore, the features determined for recognition are:

1. U-LBP features: the standard deviation and proportion of pattern 59 from 51 to 59 (two variants), symbolized as L;
2. Attributes of co-occurrence matrix: ‘Energy, ‘Correlation’ and ‘Contrast’ (three variants), symbolized as C;
3. Color Histogram Statistical Features: the average values of variance, skewness, energy, entropy in three color models (four variants), symbolized as S;
4. Ratio of the numbers of pixels between the intervals (two variants), symbolized as R.

The total number of the features chosen is 11.

### Designing classifier using SVM

SVM is a supervised learning model in machine learning, with associated learning algorithms that analyze data and recognize patterns, used for classification and regression analysis [35, 37]. It is believed that SVM is a reliable classifier superior to most traditional statistical and neural network classifiers.

In this experiment, the training dataset contains 1100 block images (100 ‘suspicious blocks’), each consisting of 11 variables, while the testing set contains 491 non-background blocks (40 ‘suspicious blocks’) from 12 WSCCIs. In the training phase, tenfold cross validation is adopted to train the model with best performance using the training set, followed by the testing phase to verify the performance of the classification using the testing set.

In our case, LIBSVM developed by Chang and Lin [38] was adopted for classification of different blocks. To find the optimal hyperplane that separates clusters of data in the feature space, parameter optimization algorithm is needed. As described below:

25 $$\begin{aligned} & {min} \quad {\frac{1}{2}\left\| {\overrightarrow {\omega } } \right\|^{T} \omega + c\sum\limits_{i = 1}^{l} {\xi_{i} } } \hfill \\& {st. }\; \quad {y_{i} (\overrightarrow {\omega }^{T} \phi (x_{i} ) + b) \ge 1 - \xi_{i} } \hfill \\ & \xi_{i} > 0,\quad \, i = 1, \ldots ,l \hfill \\ \end{aligned}$$

26 $$\text{sgn} \left( {\overrightarrow {\omega }^{T} \phi (x) + b} \right) = \text{sgn} \left( {\sum\limits_{i = 1}^{l} {\alpha_{i} } K(x_{i} ,x) + b} \right)$$

In LIBSVM, the kernel function uses radial basis function (RBF), i.e.

27 $$K(x,y) = \exp \left( {\frac{{ - \left\| {x - y} \right\|^{2} }}{{2g^{2} }}} \right)$$

**Fig. 8:**
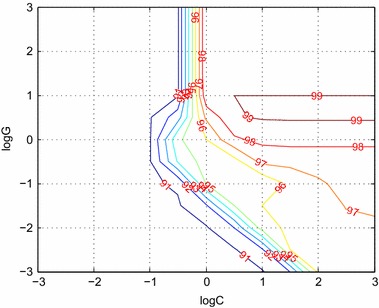
SVM parameter selection. The *horizontal axis* is the value of log c, and the *vertical axis* is the value of log g. The accuracy isocline shows in the figure

So, there are two parameters need to be specified, parameter c and parameter g. The parameters and the corresponding results are shown in Fig. 8.

The experimental procedure of SVM classification is summarized as below:

1. Normalization of the variables. To meet the requirements of LIBSVM, the variables should be normalized into values between 0 and 1. The equation is:
  28 $$X^{'} (n) = \frac{X(n) - Min}{Max - Min}$$
  where Max and Min indicates the maximal and minimum values, respectively; *X*(*n*) is the original value and $0 \le X^{'} (n) \le 1$, is the value after normalization. The Normalized variables are shown in Table 1b.

**Table 1:** Values of 11 salient variables

Features	Feature index	Quantitative variables	Mean ± SD	
			Normal (1000)	Suspicious (100)
(a) Mean and standard deviation of 11 variants of the training blocks, 1000 normal and 100 abnormal blocks				
LBP-59	1	SD	0.1188 ± 0.0171	0.0667 ± 0.0114
	2	p-59	0.0433 ± 0.0151	0.0972 ± 0.0151
Co-occurrence matrix	3	Energy	3.2022 ± 0.2686	1.8384 ± 0.4516
	4	Correlation	1.1577 ± 0.3154	0.2364 ± 0.0819
	5	Contrast	0.2087 ± 0.2672	0.9914 ± 0.5541
Statistical color histogram	6	Variance	35.1542 ± 8.8674	18.3092 ± 6.9413
	7	Skewness	0.0165 ± 0.0059	0.0036 ± 0.0032
	8	Energy	0.0160 ± 0.0061	0.0316 ± 0.0102
	9	Entropy	6.6615 ± 0.4136	5.3643 ± 0.5084
Ratio	10	R1	0.1953 ± 0.2102	0.7687 ± 0.1806
	11	R4	0.2356 ± 0.1288	0.5862 ± 0.1063

Feature index	Quantitative variables	Mean ± SD	
		Normal (1000)	Suspicious (100)
(b) Normalized mean and standard deviation of 11 variants of the training blocks, 1000 normal and 100 abnormal blocks			
1	SD	0.6659 ± 0.1632	0.1691 ± 0.1089
2	p-59	0.2441 ± 0.1457	0.7632 ± 0.1452
3	Energy	0.8435 ± 0.1096	0.2869 ± 0.1843
4	Correlation	0.5754 ± 0.1798	0.0502 ± 0.0467
5	Contrast	0.0274 ± 0.0200	0.3878 ± 0.2913
6	Variance	0.7672 ± 0.1371	0.2210 ± 0.1603
7	Skewness	0.5572 ± 0.1996	0.1075 ± 0.1088
8	Energy	0.0509 ± 0.0542	0.4057 ± 0.2552
9	Entropy	0.8540 ± 0.1004	0.4024 ± 0.2176
10	R1	0.1393 ± 0.1353	0.7013 ± 0.2397
11	R4	0.2221 ± 0.1909	0.7510 ± 0.2851

Significantly different between normal and suspicious blocks tested using t test with *p* < 0.01
2. Label the normal and suspicious blocks with positive (+1) and negative (−1) integers.
3. The dataset was two independent sets: training set for obtaining the model with best performance and testing set for verifying the performance of the SVM classifier.

## Results and discussion

### Results of background removal

As shown in Fig. 2, the difference of RUI-LBP features in gray image between background and non-background blocks is obvious, so the background blocks can be removed firstly using this difference.

Background removal is the premise of all subsequent operations, and its accuracy directly determines the feasibility of our method. 12 Cervical cell images were tested, so the total blocks are 960 (each image with 80 blocks), and the performance indices are as below:

- TP-True Positive (True Non-background Blocks)
- TN-True Negative (True Background Blocks)
- FP-False Positive (Non-background blocks judged as background)
- FN-False Negative (Background blocks judged as non-background)

Among them, the FP is the most important, so the accuracy is defined depending on FP

29 $$Accuracy = \left( {1 - \frac{FP}{80 \times n}} \right) \times 100\,\%$$

where, n-number of WSCCI needs testing. The accuracy of all the 12 tested images was 100 %, except for one image, with 1 block were FP, so the total accuracy is 99.9 %.

### Results of feature selection

After statistical analysis, only the variables that reach significant difference were chosen as the salient variables. For descriptive analysis, means and standard deviations of the 11 attributes are shown in Table 1a. With regard to ‘suspicious blocks’, the U-LBP features including standard deviation (0.0667 ± 0.0114), proportion of p-59 (0.0972 ± 0.0151); attributes of co-occurrence matrix including Energy (1.8384 ± 0.4516), Correlation (0.2364 ± 0.0819) and Contrast (0.9914 ± 0.5541); color histogram statistical features: variance (18.3092 ± 6.9413), skewness (0.0036 ± 0.0032), energy (0.0316 ± 0.0102), entropy (5.3643 ± 0.5084) and 2 ratios R2 (0.7687 ± 0.1806), R4 (0.5862 ± 0.1063) are found to be significantly different (t test, p < 0.01) with the ‘normal blocks’.

### Results of SVM classifier: independent training and testing

The 1100 non-background blocks (100 suspicious) are the training set, and tenfold cross validation were adopted to train the model with best performance. According to Fig. 8, the value of parameters c, g is 10 and 3.1623, respectively. Then another set containing 491 non-background blocks are tested to verify the performance of the classifier.

The 11 features were the input of SVM, and the output was normal and abnormal. The performance indices are calculated according to the following formula [28]:

- TP-True Positive (True abnormal blocks)
- TN-True Negative (True normal blocks)
- FP-False Positive (Normal blocks judged as abnormal blocks)
- FN-False Negative (Abnormal blocks judged as normal blocks)

30 $$Accuracy = (TP + TN)/(TP + TN + FP + FN)$$

31 $$Sensitivity = TP/(TP + FN)$$

32 $$Specificity = TN/(TN + FP)$$

33 $$PPV = TP/(TP + FP)$$

34 $$NPV = TN/(TN + FN)$$

where, PPV and NPV represent positive predictive value and negative predictive value, respectively.

Some cells were divided into different blocks, if the part was more than 50 % of the cell, the block will be trained as suspicious block.

**Fig. 9:**
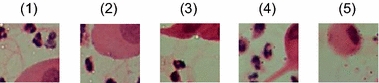
Error blocks of the algorithm. (**1**)–(**2**) are the FN blocks, (**3**)–(**5**) are the FP blocks

The classification and diagnostic performance of the SVM classifiers are shown in Table 2. The number of testing images is 12, and the number of testing blocks of non-background is 491. The testing images are different from the training images. As is shown in Table 2, the TP blocks are 38, the TN blocks are 448, the FP blocks are 3, and the FN blocks are 2, which mean that only 2 suspicious blocks are not found, and 3 normal blocks are misdiagnosed as suspicious blocks. It is very satisfying that accuracy can reach 98.98 %, and the sensitivity is 95.0 %. The error blocks are shown in Fig. 9.

**Table 2:** Results of the algorithm

Block numbers		Accuracy	
Total	960		
Background	469	Total	98.98 %
Non-background	491	Sensitivity	95.00 %
TP	38	Specificity	99.33 %
TN	448	PPV	92.68 %
FP	3	NPV	99.56 %
FN	2

### Computational complexity

Table 3 presents the processing time of the individual steps of the method. The overall processing using the Matlab code took 2.136 s on the average for 2592 × 1944 pixel images on a PC with a 3.3 GHz Intel Core i3 processor and 4 GB RAM. The running time was obtained on images having an average number of 41 non-background blocks.

**Table 3:** Comparison of average execution time with other methods of 12 testing images

	Time (s)
Step of our method	
Image resize, block-processing, background-removal	0.701
Feature selection	1.410
Classification	0.025
Total	2.136
Step of method in [16]	
Background extraction using Otsu	2.845
Candidate cell nuclei detection	62.391
Total	65.236
Step of method in [4]	
Background extraction using top-hat and thresholding	3.699
Segmentation of cells using a multi-scale hierarchical method	>100
Total	>100
Step of method in [37]	
Background extraction using MF K-means	19.737
Edge enhancement and GVF Snake	
Total	>19.737

The computational efficiency is compared with the method proposed in [11, 22, 38], in which the execution time all mentioned in their papers. To measure the efficiency, the algorithms are conducted on our images (totally 12 images, and the average time is adopted) and the comparison of the computation time is shown in Table 3. In [11] the segmentation process involves automatic thresholding to separate the cell regions from the background and a multi-scale hierarchical segmentation algorithm to partition these regions based on homogeneity and circularity. In [39], ‘Dynamic Sparse Contour Searching and GVF Snake Model’ method are used to segment partially overlapping cells; both of the methods are new and proved to work well. In [22], the authors presented a fully automated method for cell nuclei detection in Pap smear images, also without segmentation, and they detected the locations of the candidate nuclei centroids in the image with morphological. Although the method is just a premise of further recognition, it is also a new way of cervical cell detection. From Table 3, we can figure out that, without segmentation, the computational time is greatly reduced.

### Discussion

#### Block processing and background removal

As the first step of this algorithm, acquisition of block images is of great importance. The resolution of the original image and the size of the block image both have certain effects on the final accuracy. Higher resolution and higher definition can provide more detail features for images, but the large amount of data makes the following intelligent processing more difficult. Therefore, in this study, with the original size 2592 × 1000, the images are down sampled to 800 × 1000 for little calculation while preserving the basic features of the images. The size of the block is also critical. If the size is too large, there may be too many cells in one block, then the block processing will be meaningless. If the size is too small, the cells may be divided into too many parts, then the features of the cells will be inaccurate. Therefore, in this study, the images are divided into blocks with size 100 × 100, which is larger than one whole suspicious cell, but smaller than two complete suspicious cells. The resolution of the images and the size of the blocks still remain an issue on which should be studied further.

During the analysis of block image features, the RIU-LBP distribution of background blocks is very different from the non-background blocks, which inspired us to remove the background blocks first. With this step, as illustrated in section “Computational complexity”, about half of the blocks are removed, therefore, the computational time is reduced by 1 time.

The primary goal of these two procedures is to increase computational speed. Table 3 compares the computational time of the block image processing method in this study with other segmentation methods. As indicated in this table, block processing is more efficient than the segmentation methods. Therefore, the clinical application of block-processing method is very optimistic for real-time screening.

#### Feature analysis and block classification

Traditional criteria for differentiating dysplastic cells from normal cells are based on the ratio of nucleus to cytoplasm (N/C ratio), nuclear size, nuclear shape, and density and granularity of nuclear chromatin. Most of these criteria are subjective, relatively. In contrast, in computer aided diagnosis systems, the subjective criteria are replaced by quantitative, calculable variables [22]. In this study, texture and color features of three color models are analyzed. Texture features including LBP features and co-occurrence matrix features are calculated, while color features including statistical histogram color features and ratios of color intervals are calculated.

In this study, tenfold cross validation is adopted to train the model using the training set, and 11 texture and color variables with the highest accuracy are selected as salient features. The features are categorized into four groups, including (1) U-LBP features (standard deviation, value of p-59); (2) attributes of co-occurrence matrix (Energy, Correlation and Contrast); (3) color histogram statistical features (variance, skewness, energy, entropy); and (4) two ratios of color intervals (R2, R4). By taking individual variables into consideration, the statistical results (Table 1a) show that the 11 selected variables are able to differentiate ‘suspicious blocks’ from ‘normal blocks’ (p < 0.01, t test). It is indicated that the U-LBP distribution of ‘suspicious blocks’ is more uniform (lower standard deviation), and has higher irregularity (greater contrast, lower energy and correlation). The color average value of the ‘suspicious blocks’ is neither too low (as the ‘many-white’ and ‘clustered white’) nor too high (as the ‘few-white’ and ‘normal block’). As for color distribution, the histogram of ‘suspicious blocks’ is more uniform, concentrated in a smaller range compared with ‘many-white’, ‘clustered white’ and ‘normal’. Therefore, the ‘variance’, ‘skewness’ and ‘entropy’ values are lower, while ‘energy’ is higher. *R*_2_, *R*_4_ are higher in ‘suspicious block’, which indicates that the nuclear ratio are higher in ‘suspicious block’.

As for the classification, as indicated in Table 2, the total accuracy of this method is 98.98 %, which means that only 5 out of 491 blocks are misdiagnosed, in which the FN number is 2, and the FP number is 3. In our study, we deal with the whole image instead of one cell, which makes it more useful for clinical applications.

## Conclusion

Differing from conventional methods, the approach is a new thought for cervical cell image screening, avoiding image segmentation, which is a tough task in cell image processing. To the best of our knowledge, there are few works finding abnormal cells from the whole cervical cell image, so our work is of great significance to some extent, and might be really useful in clinical applications. In our case, for the testing data, the sensitivity and accuracy are satisfying. Therefore, the algorithm will be effective in the preliminary screening. Although some analysis work should be done in advance, with the model we built, the screening will be much faster than the method with segmentation.

There are still some problems need to be studied, such as how to choose the block size. Another problem is that since the SVM model is only for H&E staining images in this algorithm, more work should be done to apply to a wider range of images. More subsequent investigation with statistical measurements is needed to elucidate its practical utility in a laboratory and its ability to improve the diagnostic performance of a laboratory.
